# Expression of a germline variant in the N-terminal domain of the human DNA glycosylase NTHL1 induces cellular transformation without impairing enzymatic function or substrate specificity

**DOI:** 10.18632/oncotarget.27548

**Published:** 2020-06-16

**Authors:** Carolyn G. Marsden, Pawel Jaruga, Erdem Coskun, Robyn L. Maher, David S. Pederson, Miral Dizdaroglu, Joann B. Sweasy

**Affiliations:** ^1^Department of Microbiology and Molecular Genetics, The Markey Center for Molecular Genetics, University of Vermont, Burlington, VT 05405, USA; ^2^Biomolecular Measurement Division, National Institute of Standards and Technology, Gaithersburg, MD 20899, USA; ^3^Department of Cellular and Molecular Medicine, University of Arizona Cancer Center, Tucson, AZ 85724, USA; ^4^Present address: Saint Michael’s College, Colchester, VT 05439, USA; ^5^Present address: Institute for Bioscience and Biotechnology Research, University of Maryland, Rockville, MD 20850, USA

**Keywords:** NTHL1, cellular transformation, germline variant, base excision repair

## Abstract

Oxidatively-induced DNA damage, widely accepted as a key player in the onset of cancer, is predominantly repaired by base excision repair (BER). BER is initiated by DNA glycosylases, which locate and remove damaged bases from DNA. NTHL1 is a bifunctional DNA glycosylase in mammalian cells that predominantly removes oxidized pyrimidines. In this study, we investigated a germline variant in the N-terminal domain of NTHL1, R33K. Expression of NTHL1 R33K in human MCF10A cells resulted in increased proliferation and anchorage-independent growth compared to NTHL1 WT-expressing cells. However, wt-NTHL1 and R33K-NTHL1 exhibited similar substrate specificity, excision kinetics, and enzyme turnover *in vitro* and *in vivo*. The results of this study indicate an important function of R33 in BER that is disrupted by the R33K mutation. Furthermore, the cellular transformation induced by R33K-NTHL1 expression suggests that humans harboring this germline variant may be at increased risk for cancer incidence.

## INTRODUCTION

Maintenance of genomic integrity requires proper functioning of the base excision repair (BER) pathway, which is responsible for the repair of at least 30,000 lesions per cell per day arising from endogenous insults [1, 2]. BER is initiated when a DNA glycosylase locates a lesion and catalyzes the cleavage of the N-glycosyl bond between the base and 2’-deoxyribose generating an AP (apurinic/apyrimidinic) site. Bifunctional DNA glycosylases, which generally repair oxidatively-induced DNA lesions, possess both glycosylase and lyase activity. Therefore, bifunctional DNA glycosylases can potentially cleave the DNA backbone (utilizing their lyase activity) generating DNA ends that are further processed by AP endonuclease I (APE1) or polynucleotide kinase (PNK). Insertion of the missing base by DNA polymerase β (Pol β) and sealing of the nick by X-Ray Cross-Complimenting 1 (XRCC1) complexed with DNA ligase I or IIIα completes repair [2, 3]. Consisting of only four-five reaction steps, BER has traditionally been considered one of the simplest DNA repair pathways; however, recent studies have begun to unveil the complexities of BER [2]. Although BER can be reconstituted *in vitro* by combining the enzymes necessary for each step in the pathway, *in vivo* studies have begun to unveil the complex interplay between BER enzymes and other repair and non-repair proteins driving accurate and efficient repair in the cellular environment.

NTHL1, the mammalian homolog of *Escherichia coli* (*E. coli*) endonuclease III, is a bifunctional DNA glycosylase that primarily recognizes and removes a wide range of modified pyrimidine derivatives, and purine-derived 4,6-diamino-5-formamidopyrimidine (FapyAde) and 2,6-diamino-4-hydroxy-5-formamidopyrimidines (FapyGua) from DNA [2, 4–12]. The core catalytic regions of NTHL1, including the Lys 220 and Asp 239 catalytic residues, as well as the iron-sulfur binding cluster (4Fe-4S cluster loop) are highly conserved with its bacterial homolog. However, mammalian NTHL1 contains a non-conserved N-terminal domain of 95 amino acids that is predicted to be disordered [11]. Despite conservation of catalytic domains/residues and DNA-binding motifs between NTHL1 and *E. coli* endoIII, NTHL1 has been shown to differ in substrate specificity and excision kinetics from its bacterial homologues [5, 13, 14]. Truncation of the N-terminal domain up to 80 amino acids has been shown to increase product release and enzyme turnover without impacting glycosylase or AP lyase activities [15]. Collectively, the evidence suggests a negative regulatory role of the N-terminal domain, possibly preventing premature product release by NTHL1 and exposure of cytotoxic and mutagenic BER intermediates. Interestingly, there are many DNA glycosylases and BER proteins that contain regions of disorder that are not conserved in their prokaryotic orthologs, including NEIL1, NEIL2, OGG1, MPG, APE1 and Pol β reviewed in [16]. Disordered domains are presumed to function in protein: protein and protein: DNA interactions given their inherent structural flexibility and plasticity, however the functions of these disordered domains have yet to be elucidated. Similarly, the functions of the N-terminal disordered region regarding NTHL1 regulation, BER coordination, various cellular processes, or genomic stability remains entirely unknown.

Several studies have linked alterations of NTHL1 function with carcinogenesis. Double-knockout *Nthl1*^-/-^ */Neil1*^-/-^ mice have a higher incidence of pulmonary and hepatocellular tumors compared to single-knockout strains, which may have been associated with accumulation of FapyAde and FapyGua [7], which are mutagenic, with FapyGua being even more mutagenic than 8-hydroxyguanine (8-OH-Gua) [17–20]. Mislocalization of NTHL1 to the cytoplasm as well as single nucleotide polymorphisms (SNPs) in the promoter region of NTHL1, resulting in decreased expression, have been linked to the etiology of a subset of human gastric tumors [21]. Two independent research groups have identified a germline nonsense mutation in NTHL1 in patients with multiple primary tumors, including colorectal cancer [22, 23]. The broad increase in tumor incidence in those patients harboring the NTHL1 nonsense mutation established a novel “NTHL1 syndrome” [23, 24]. More recently, work from our laboratory showed that expression of a germline variant that results in mutation of aspartic acid (D) 239 to tyrosine (Y) in NTHL1 induced cellular transformation and genomic instability in human non-transformed epithelial cells [4]. These studies demonstrate the inextricable link of NTHL1 dysfunction with cancer, providing impetus for investigations into the potential impact of additional germline mutations in the gene encoding for NTHL1 on genome integrity and cellular transformation.

In this study, we have characterized a missense germline mutation that results in the mutation of arginine 33 to lysine (R33K) (dsSNP: rs2302172) in the N-terminal domain of NTHL1. Although predicted to be non-damaging, expression of R33K-NTHL1 in MCF10A cells results in cellular transformation without impairing enzymatic function or substrate specificity. Collectively, our data provide evidence that expression of a conservative single amino acid mutation in the N-terminal domain of NTHL1 has significant deleterious effects on vital cellular processes, independent of its catalytic functions. These findings begin to elucidate the critical functions of the N-terminal domain in NTHL1 and further underscore the need for continuous investigations into the impact of germline mutations in genes that encode for BER proteins on genome integrity.

## RESULTS

### Expression of NTHL1 R33K in a human epithelial cell line induces cellular transformation

**Figure 1:**
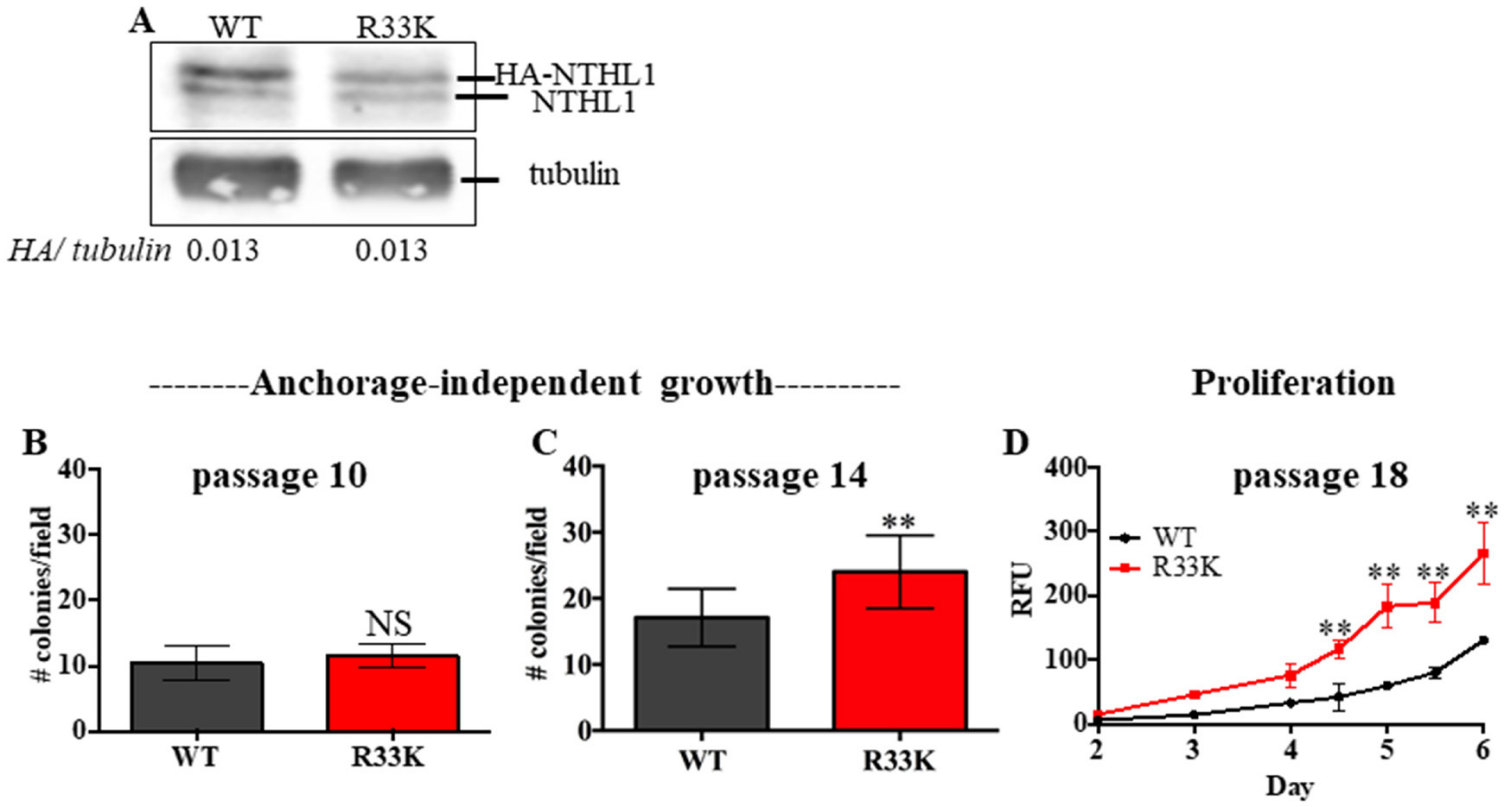
Expression of R33K-NTHL1 induces cellular transformation. (**A**) Western blot showing equivalent expression of exogenous HA-tagged wt-NTHL1 and HA-tagged R33K-NTHL1. Image has been cropped and contrast/brightness has been adjusted for both channels for visualization purposes only. Quantitation of bands performed using data embedded in image file at time of collection. (**B**–**D**) Expression of NTHL1 R33K in MCF10A cells increases anchorage independent growth (B, C) and proliferation (D) indicative of cellular transformation. Anchorage independent growth (B, C): Data are graphed as mean colonies/field. Uncertainties are standard deviations (SD). ^**^ *p* < 0.01. Proliferation (D): Data are graphed as mean relative fluorescent units (RFU). Uncertainties are standard deviations. ^**^ *p* < 0.01.

**Figure 2:**
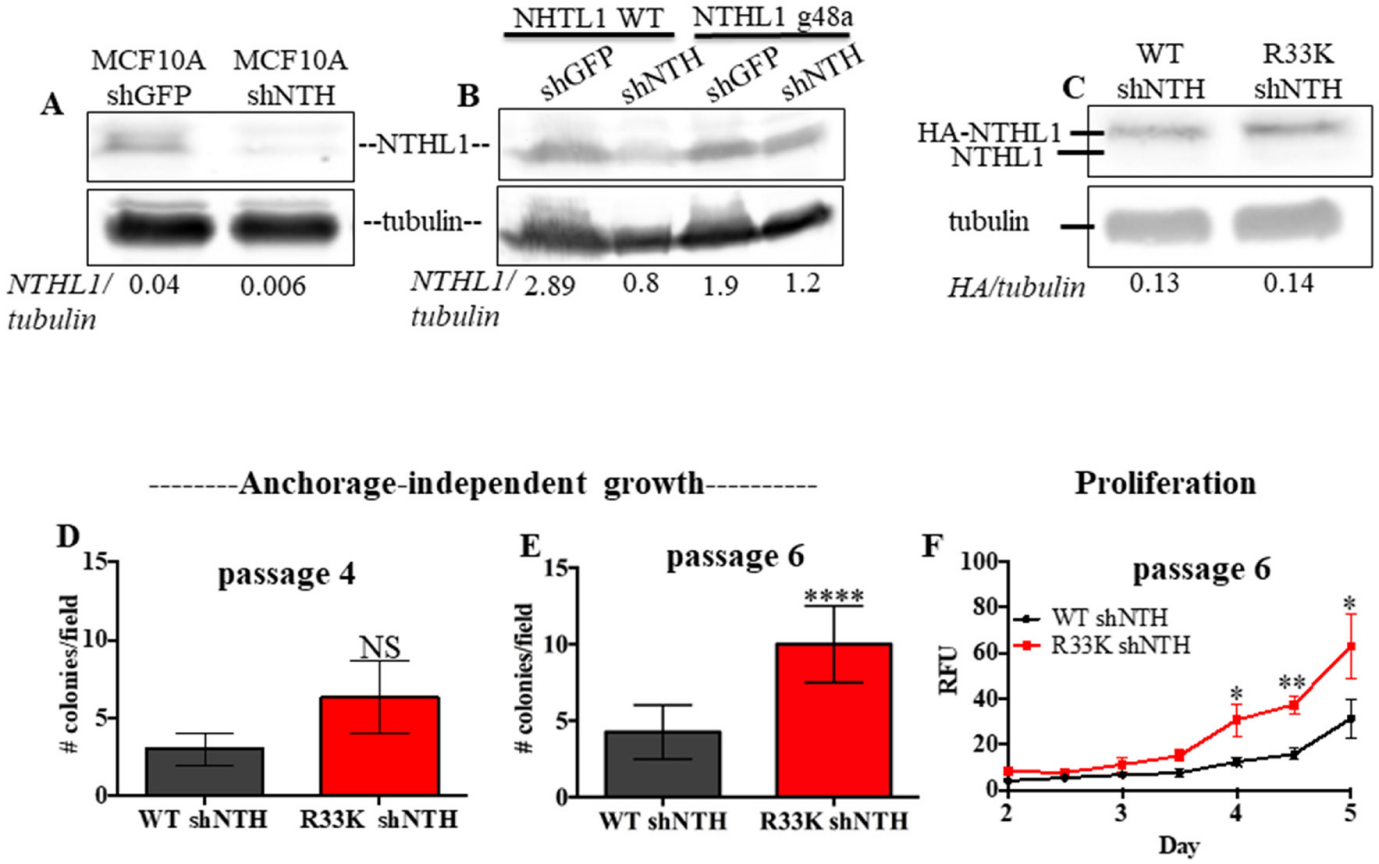
Expression of R33K-NTHL1 with knockdown of endogenous NTHL1 accelerates cellular transformation. (**A**) Western blot demonstrating that shRNA expression constructs effectively knockdown endogenous NTHL1 in MCF10A cells. Image has been cropped and contrast/brightness has been adjusted for both channels for visualization purposes only. Quantitation of bands performed using data embedded in image file at time of collection. (**B**) Western blot demonstrating that NTHL1 g48a construct is resistant to targeting by overlapping shRNA constructs with overlapping sequence homology in NTHL1 (binding to similar sequence in NTHL1) and shGFP construct does not reduce exogenous or endogenous NTHL1 expression. Image has been cropped and contrast/brightness has been adjusted for both channels for visualization purposes only. Quantitation of bands performed using data embedded in image file at time of collection. (**C**) Western blot showing equivalent expression of exogenous HA-tagged wt-NTHL1 (g48a) and HA-tagged R33K-NTHL1 (g48a) and knockdown of endogenous NTHL1 in MCF10A pools with stable expression of HA-tagged wt- or R33K-NTHL1. Image has been cropped and contrast/brightness has been adjusted for both channels for visualization purposes only. Quantitation of bands performed using data embedded in image file at time of collection. (**D**–**F**) Expression of R33K-NTHL1 in MCF10A cells increases anchorage independent growth (D, E) and proliferation (F) at passage 6, indicative of cellular transformation. Anchorage independent growth (D, E): Data are graphed as mean colonies/field. Uncertainties are standard deviations (SD). ^****^ *p* < 0.0001. Proliferation (F): Data are graphed as mean relative fluorescent units (RFU). Uncertainties are standard deviations. ^*^ *p* < 0.05; ^**^ *p* < 0.01.

**Figure 3:**
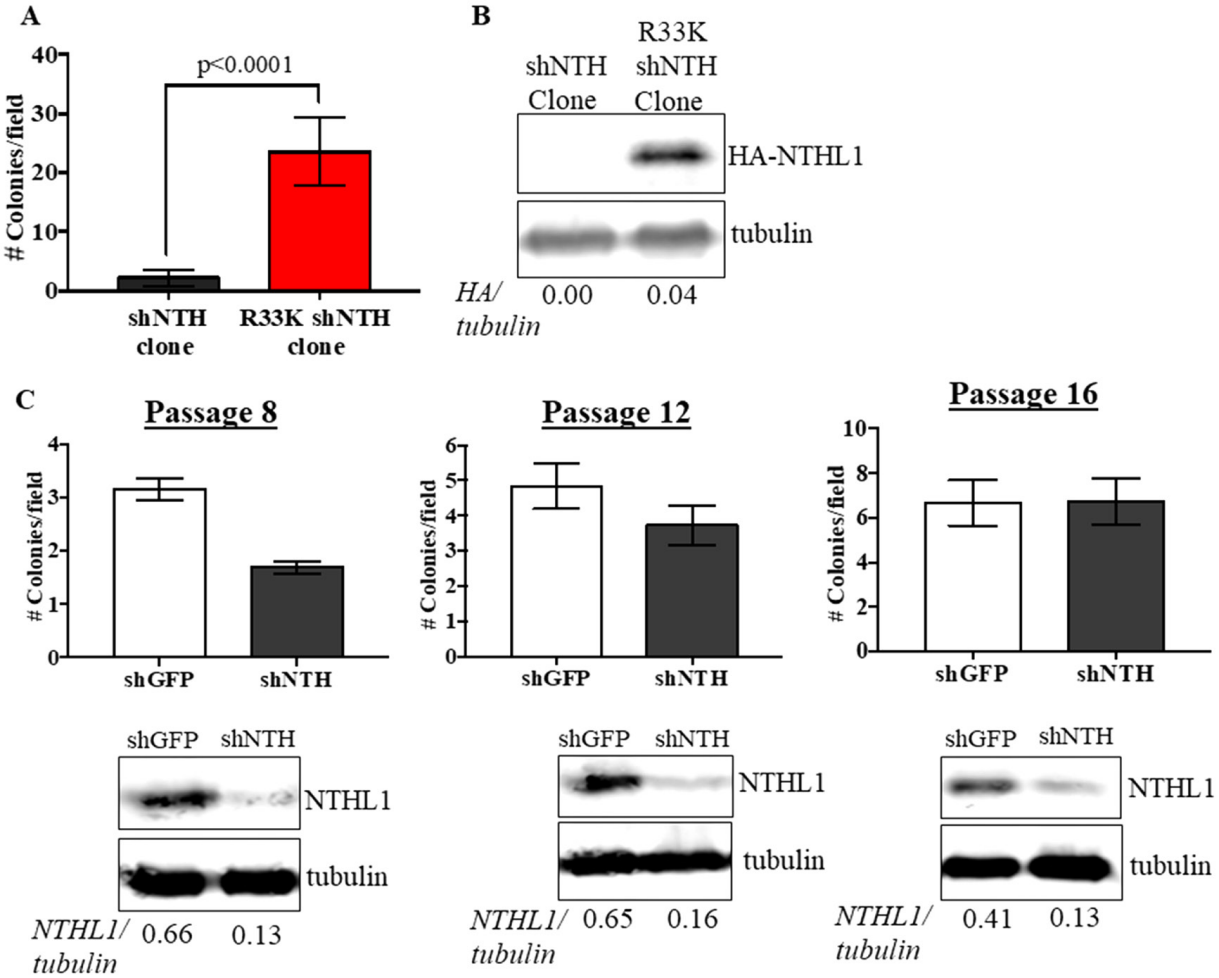
Expression of R33K-NTHL1 is required for cellular transformation. (**A**) Knockdown (shNTH) cell populations derived from R33K-NTHL1 clone that express NTHL1 R33K generated significantly more colonies as compared to knockdown (shNTH) cell populations derived from shNTH clone that did not express detectable levels of R33K-NTHL1. Data are graphed as mean colonies/field. Uncertainties are standard deviations (SD). (**B**) Western blot showing no detectable expression of R33K-NTHL1 in shNTH clone and expression of R33K-NTHL1 in cell populations derived from R33K-NTHL1 clone. Image has been cropped and contrast/brightness has been adjusted for both channels for visualization purposes only. Quantitation of bands performed using data embedded in image file at time of collection. (**C**) Knockdown of NTHL1 does not result in increased levels of cellular transformation in MCF10A cells. Data are graphed as mean colonies/field. Uncertainties are standard deviations (SD). Western blots demonstrate maintenance of NTHL1 knockdown at passages tested for anchorage-independent growth.

To determine the cellular effects of R33K-NTHL1 expression, we generated MCF10A cells (a non-transformed human breast epithelial cell line) with stable and equivalent expression of HA-tagged wt-NTHL1 or HA-tagged R33K-NTHL1 as previously described (Figure 1A) [4]. As a human germline mutation, we were first interested in testing whether R33K-NTHL1 expression in MCF10A cells could induce phenotypes indicative of cellular transformation. Therefore, we measured proliferation (using the CyQUANT NF Cell Proliferation Assay) and anchorage-independent growth (using the CytoSelect 96-well cell transformation Assay) of wt-NTHL1 and R33K-NTHL1 pools. As shown in Figure 1B–1D expression of R33K-NTHL1 resulted in increased anchorage independent growth and increased proliferation as compared to wt-NTHL1-expressing cells. R33K-NTHL1-expressing cells exhibited increased anchorage-independent growth by passage 14 (Figure 1B, 1C) and increased proliferation by passage 18 (Figure 1D). The highly conservative nature of the mutation (Arg → Lys), which maintains overall bulkiness and charge at amino acid position 33, prompted the additional generation of MCF10A HA-tagged wt-NTHL1 or HA-tagged R33K-NTHL1-expressing cells with knockdown of endogenous NTHL1 (wt/R33K-NTHL1 shNTH) to reduce possible compensation from the endogenous protein and consequent masking of potential cellular phenotypes. Two short-hairpin RNAs (shRNA) with similar sequence homology to the target sequence in NTHL1 (with binding sites in NTHL1 that overlap) were utilized to increase the efficacy of knockdown and provide an additional level of control of expression. The shRNAs were stably expressed in MCF10A cells and expression of endogenous NTHL1 was measured by Western blot. As shown in Figure 2A, expression of endogenous NTHL1 was efficiently reduced. To achieve knockdown of endogenous NTHL1 without impacting expression of exogenously expressed wt-NTHL1 or R33K-NTHL1, two silent mutations were generated in the open reading frame (ORF) of wt-NTHL1 and R33K-NTHL1 (labeled as NTHL1 g48a) within the sequence targeted by two overlapping shRNAs (Figure 2B). Therefore, the exogenous NTHL1 proteins were resistant to silencing by the shRNAs while achieving efficient knockdown of endogenous NTHL1 protein expression (Figure 2B, labeled herein as wt-NTHL1 shNTH or R33K-NTHL1 shNTH). As with the dominant pools, we measured proliferation and anchorage-independent growth of wt-NTHL1 shNTH R33K-NTHL1 shNTH pools beginning at passage 2 with equivalent expression of exogenous HA-tagged wt-NTHL1 or R33K-NTHL1 (Figure 2C). As shown in Figure 2D–2F, expression of R33K-NTHL1 shNTHL1 resulted in increased anchorage independent growth and increased proliferation, respectively, as compared to wt-NTHL1-expressing cells. R33K-NTHL1 shNTH-expressing cells exhibited increased anchorage independent growth (Figure 2E) and proliferation (Figure 2F) as early as passage 6. Furthermore, we show that expression of NTHL1 R33K is required for increased anchorage-independent growth. Importantly, shNTH clonal cell lines with R33K-NTHL1 expression exhibit significantly increased levels of colonies growing in an anchorage independent manner when compared to shNTH cells that do not express R33K-NTHL1 (Figure 3A, 3B). Notably, knockdown of endogenous NTHL1 alone does not induce cellular transformation up to passage 16 (Figure 3C). Therefore, the expression of R33K-NTHL1 induces cellular transformation, which is significantly accelerated in the absence of endogenous NTHL1.

### NTHL1 R33K does not alter cellular localization

**Figure 4:**
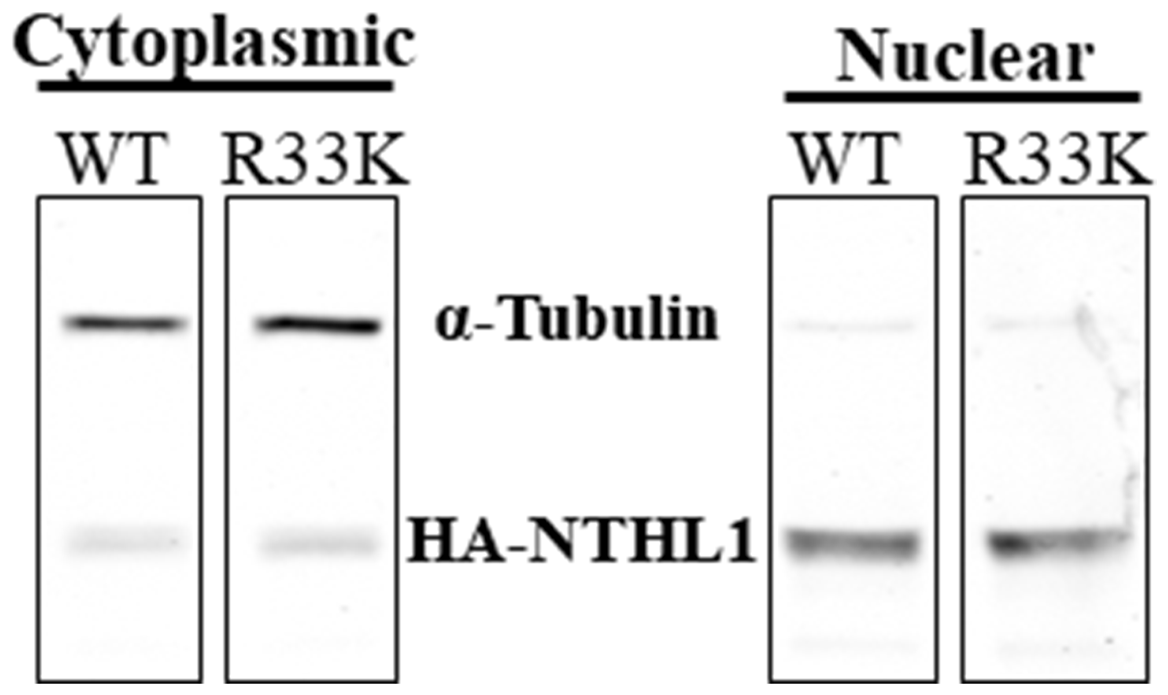
R33K does not impact cellular localization. Fractionation of cytoplasmic and nuclear compartments in MCF10A wt-NTHL1 or R33K-NTHL1 shNTH cell lysates demonstrates nuclear localization of wt- and R33K-NTHL1. Nuclear localization of wt-NTHL1 serves as a positive control for nuclear localization of R33K-NTHL1. Image was obtained using an Odyssey CLx Infrared Imaging System. Fluorescent signals were measured using a 700 Channel laser source (solid state diode laser at 785 nm) and 800 Channel laser source (solid state diode laser at 785 nm). Band intensities were quantified using the data recorded during image capture using Image Studio™ Software (LI-COR).

The R33K germline mutation is located between the mitochondrial and nuclear localization signals in the N-terminal domain of NTHL1. Therefore, we tested whether cellular localization of NTHL1 is disrupted by the R33K mutation by preparing cytoplasmic and nuclear extracts then blotting for NTHL1 and alpha tubulin (as a control for separation of the cellular compartments). As shown in Figure 4, wt-NTHL1 and R33K-NTHL1 exhibit similar cellular localization. These data demonstrate that R33K has no impact on cellular localization of NTHL1 and therefore the transformative phenotypes observed in R33K-NTHL1 shNTH-expressing cells cannot be attributed to changes in cellular localization.

### NTHL1 R33K does not impact enzymatic functions

**Figure 5:**
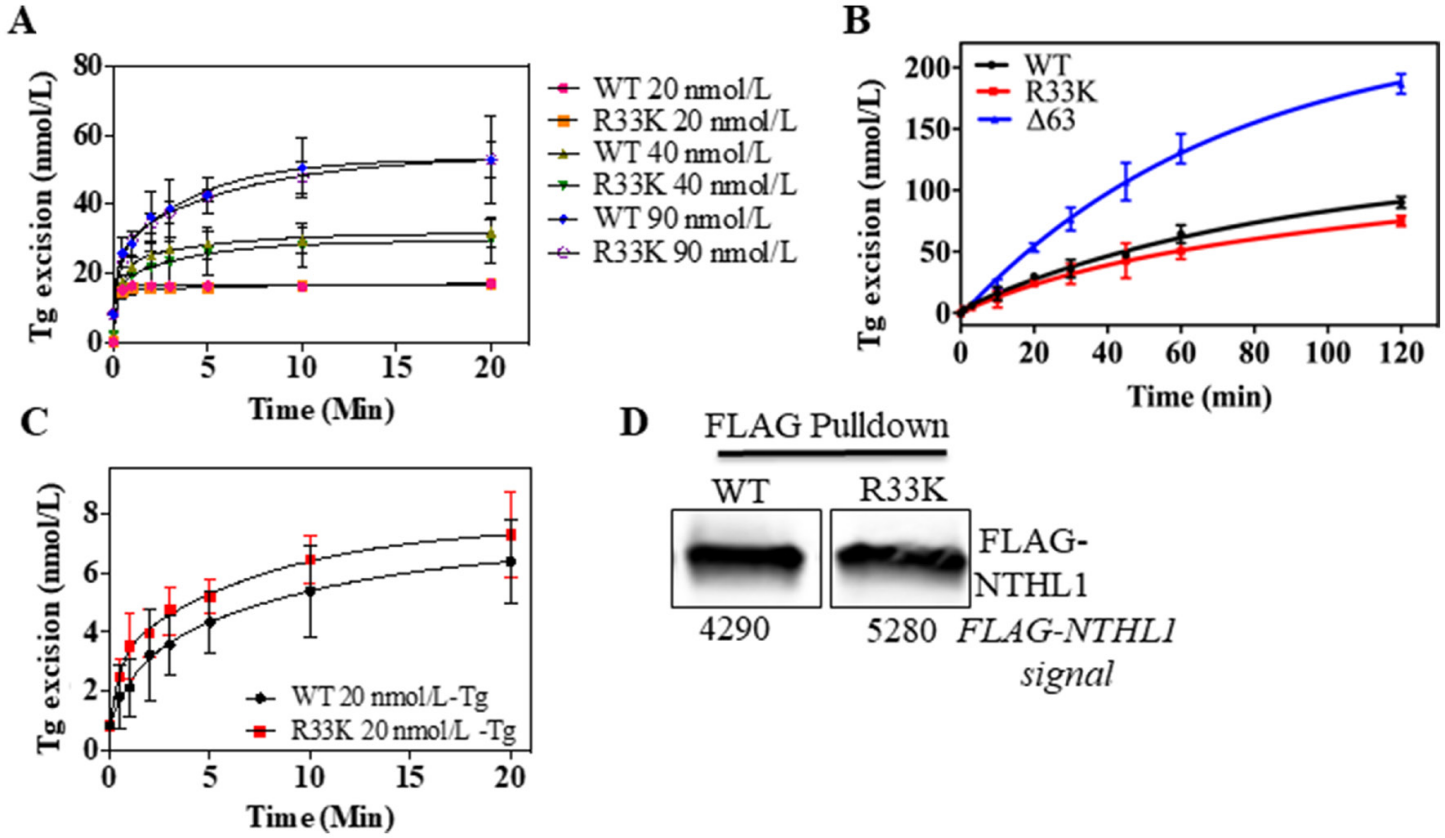
Wt- and R33K-NTHL1 proteins exhibit similar enzyme kinetics. (**A**) Excision activity of bacterially-expressed wt-NTHL1 and R33K-NTHL1 proteins (10 nmol/L) of a ThyGly lesion (20–90 nmol/L) demonstrates equivalent excision kinetics of wt-NTHL1 and R33K-NTHL1 under multiple turnover conditions. (**B**) Multiple turnover excision kinetics demonstrate that the R33K mutation does not impact enzymatic turnover of bacterially-expressed R33K-NTHL1 (10 nM) as compared to bacterially-expressed Δ63-NTHL1 (10 nmol/L) which exhibits increased enzymatic turnover (positive control). (**C**, **D**) Multiple turnover kinetics using FLAG purified proteins from HEK293T transfected cells indicates similar enzymatic activity between wt- and R33K-NTHL1 expressed in a human cell line. Western blot demonstrates mostly equivalent amounts of FLAG purified WT and R33K protein. Data are graphed as Tg excision (nmol/L). Uncertainties are standard deviations (SD).

**Figure 6:**
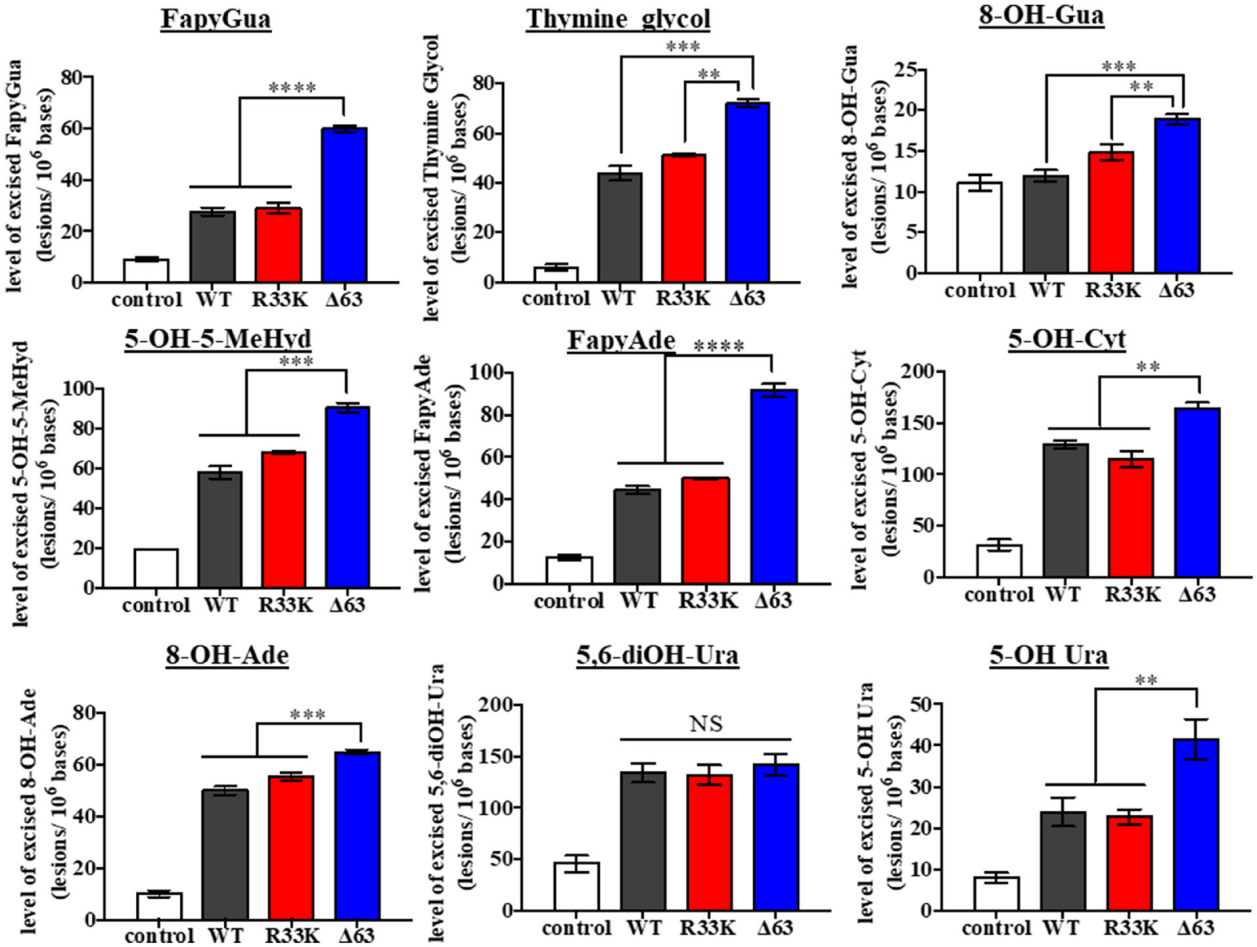
Wt- and R33K-NTHL1 exhibit similar substrate specificity. Levels of DNA base lesions excised from irradiated calf thymus DNA by wt-NTHL1, R33K-NTHL1, or Δ63-NTHL1. Control = no enzyme. Data are graphed as the level of excised base lesions. Uncertainties are standard deviations (SD).

Although previous studies have shown that the N-terminal domain (up to 80 amino acids) is dispensable for glycosylase/lyase functions [15], we tested whether R33K-NTHL1 disrupts excision of ThyGly from a double-stranded oligodeoxynucleotide (dsOligo) substrate under multiple turnover conditions using bacterially-expressed wt-NTHL1 and R33K-NTHL1. As expected, bacterially-expressed R33K-NTHL1 exhibited excision kinetics equivalent to that of wt-NTHL1 (Figure 5A). A previous study showed that truncation of the N-terminal domain (up to 80 amino acids) decreases product inhibition of NTHL1, leading to increased enzymatic turnover [15]. Given the location of R33K in the N-terminal domain of NTHL1, we tested whether this mutation altered product inhibition similar to the N-terminal truncation mutant, Δ63-NTHL1. As show in Figure 5B, R33K-NTHL1 does not increase enzyme turnover as compared to Δ63-NTHL1, included as a positive control. We also tested for differences in enzymatic activity of DYKDDDDK tag (FLAG)-purified wt-NTHL1 and R33K-NTHL1 from human embryonic kidney cells (HEK293T cells) using a dsOligo ThyGly-substrate. However, there were no differences in excision kinetics detected between FLAG-purified wt-NTHL1 and R33K-NTHL1 proteins (Figure 5C, 5D). To test for changes in substrate specificity, γ-irradiated calf thymus DNA (containing a broad range of DNA lesions) was incubated with bacterially-expressed wt-NTHL1, R33K-NTHL1 or Δ63-NTHL1, and the number of DNA base lesions excised from the DNA were identified and quantified by GC-MS/MS. Wt-NTHL1 and R33K-NTHL1 excised similar levels of nine DNA base lesions (Figure 6), exhibiting similar substrate specificities. These results are consistent with the results presented in Figure 5B, showing similar excision kinetics of ThyGly by wt-NTHL1 and R33K-NTHL1. No significant excision of 8-OH-Gua was observed in agreement with previous results [5, 6]. The greater excision of most of the lesions by Δ63-NTHL1 than the excision by wt-NTHL1 or R33K-NTHL1 is on a par with the data in Figure 5B in terms of the greater excision of ThyGly by Δ63-NTHL1 from the dsOligo ThyGly-substrate than the excision by wt-NTHL1 or R33K-NTHL1.

### R33K-NTHL1 does not impact enzymatic functions *in vivo* under normal or oxidatively-stressed conditions

**Figure 7:**
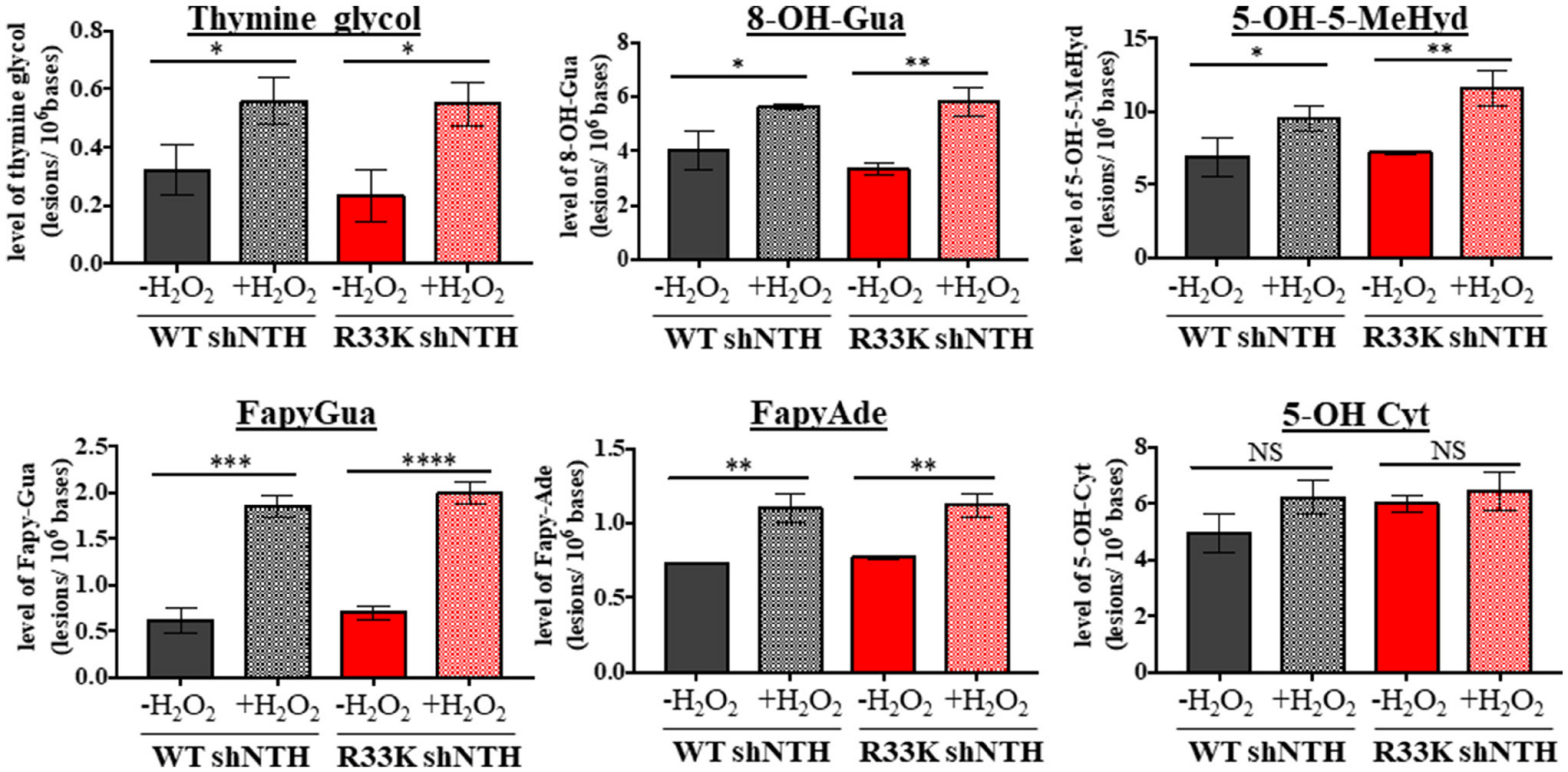
Wt- and R33K-NTHL1 exhibit similar substrate specificity and repair kinetics *in vivo*. Levels of DNA base lesions in genomic DNA isolated from unstressed or oxidatively-stressed (10 mmol/L H_2_O_2_ treatment for 30 min followed by 30 min recovery) wt-NTHL1 or R33K-NTHL1 shNTH- expressing MCF10A cells. Data are graphed as the level of excised base lesions. Uncertainties are standard deviations (SD).

It has been proposed that initial scanning by DNA glycosylases for DNA base lesions may be mediated by electrostatic, non-specific and mostly transient interactions between the DNA and disordered domains present in eukaryotic DNA glycosylases, given the inherent flexibility of disordered regions as well as the clustering of positively charged and basic residues (as seen in the disordered domain of NTHL1) [for a review see [16]]. Therefore, the R33K mutation may disrupt the scanning behavior of NTHL1 within the context of the cell. Additionally, DNA in the cell is organized into chromatin which can impede access to lesions by DNA glycosylases and thus inhibit repair. However, lesion recognition and excision kinetics has thus far been tested on dsOligos and de-proteinized calf thymus DNA. To test this hypothesis, asynchronous MCF10A wt-NTHL1- and R33K-NTHL1 shNTH-expressing cells were treated with 10 mmol/L H_2_O_2_. Genomic DNA was isolated from control and treated cells, and DNA base lesions were identified and quantified by GC-MS/MS. As Figure 7 shows, the background levels and oxidatively-induced levels of each of the six base lesions were similar in both untreated wt-NTHL1 and R33K- NTHL1 shNTH-expressing cells (no significant differences detected). Following the H_2_O_2_-treatment, significant increases in the levels of DNA lesions were observed (except for in the level of 5-OH-Cyt) in wt-NTHL1 and R33K-NTHL1 shNTH- expressing cells. The levels of 8-OH-Ade and 5,6-diOH-Ura were also measured; however, no increases in their levels were observed over the background levels in either cell lines (data not shown). These results indicate that R33K-NTHL1 does not disrupt DNA scanning, initial lesion recognition, or lesion excision by NTHL1 broadly *in vivo*. They are also on a par with the results shown in Figure 6. Collectively, these data demonstrate that R33K has no impact on enzymatic function or substrate specificity of NTHL1 and therefore the transformative phenotypes observed in R33K-NTHL1 shNTH-expressing cells cannot be attributed to disruption of enzymatic repair functions.

## DISCUSSION

In this study, we have shown that expression of the NTHL1 germline variant, R33K-NTHL1, in MCF10A cells induces cellular transformation as measured by increased proliferation and increased anchorage-independent growth. Investigations into the molecular mechanisms driving cellular transformation in R33K-NTHL1 shNTH-expressing cells indicate that R33K mutation does not impact NTHL1 enzymatic functions, substrate specificity or coordination of BER in the cell. The cellular transformation induced by the expression of R33K-NTHL1 implicate a potential role for this germline mutation in the initiation of human cancer and therefore individuals harboring this mutation may be at a higher risk for lifetime cancer incidence.

Mutation of arginine 33 to a lysine is a highly conservative amino acid change, maintaining charge and overall bulkiness at the residue. Folding or stability of the protein would not be impacted given the location of R33K in the N-terminal domain of NTHL1, which is predicted to be disordered (Polyphen). As we have shown in this study, the R33K mutation has no observable impact on glycosylase/lyase functions or substrate specificity. This is not altogether surprising given a previous study that showed truncation of the N-terminal domain of NTHL1 (up to 80 amino acids) had no effect on glycosylase/lyase activity but did increase product release and enzyme turnover, thus resulting in enhanced activity under enzyme limiting conditions [15]. Although located in the N-terminal domain, R33K-NTHL1 does not increase enzyme turnover as observed with Δ63-NTHL1. Despite no observed changes in enzymatic activity or substrate recognition/specificity, expression of R33K-NTHL1 in MCF10A cells results in increased proliferation and anchorage-independent growth which are indicative of cellular transformation. Therefore, the question remains how a highly conservative mutation in the disordered domain of NTHL1 that is dispensable for its enzymatic functions induces cellular transformation. The disordered region in NTHL1 is not conserved with its bacterial homologue, which suggests an evolutionary adaptation for the complexity of repair mechanisms in mammalian systems. NTHL1 has been shown to differ in substrate specificity and excision kinetics from its bacterial homolog [5, 6, 13, 14]. As previously discussed, the N-terminal domain in NTHL1 was shown to decrease enzyme turnover *in vitro* [15]. These studies suggest that the N-terminal domain of NTHL1 negatively regulates NTHL1 function, however no studies to date have elucidated the functions of the N-terminal domain of NTHL1. In fact, our study provides the first evidence for a critical role of the N-terminal domain in NTHL1 in cellular processes that prevent cellular transformation. Therefore, characterization of NTHL1 as a tumor suppressor does not depend only on the catalytic functions (glycosylase/lyase activities) but also on the functions of the N-terminal disordered region. One likely function of the disordered N-terminal domain in NTHL1 is to serve as an interface for interactions with other BER and non-BER proteins. One possible hypothesis we propose is that R33 is a key amino acid for specific protein interactions and R33K may disrupt those NTHL1-protein interactions resulting in altered NTHL1 function and the cellular outcomes observed. Investigations are ongoing to identify potential NTHL1-protein interactions by the R33K mutation.

## MATERIALS AND METHODS

### Plasmids, cloning and recombinant proteins

A short hairpin RNA (shRNA)-resistant human wt-NTHL1 vector (referred to as NTHL1 g48a) was generated by introducing two silent mutations [25] using site-directed mutagenesis within the overlapping binding regions of TRC human NTHL1 shRNA constructs (CloneID: TRC0000007915 and CloneID: TRC0000007916) (Dharmacon). NTHL1 R33K was generated using standard methods of site-directed mutagenesis using the shRNA-resistant wt-NTHL1 g48a construct as a template. Wt-NTHL1 and R33K-NTHL1 were expressed and purified from *E.coli* as previously described [4].

### Cell lines and cell culture

GP2-293 cells (Clonetech) and HEK293T cells were maintained in Dulbecco’s Minimal Essential Medium (DMEM) (Corning, Cellgro) 10% fetal bovine serum (FBS) (Invitrogen) supplemented with 1% penicillin-streptomycin. MCF10A cells, an immortalized, non-transformed mammary epithelial cell line, were obtained from American Type Culture Collection (ATCC). MCF10A cells were maintained in Dulbecco’s Modified Eagle Medium: Nutrient Mixture F-12 (DMEM/F12) medium (Corning, Cellgro) supplemented with 5% horse serum (HyClone), 1% penicillin-streptomycin (Gibco), epidermal growth factor (EGF) (20 ng/mL, Peprotech), hydrocortisone (0.5 μg/mL, Sigma-Aldrich), insulin (10 μg/mL, Invitrogen), Cholera Toxin (100 ng/mL, Sigma-Aldrich). All cells were maintained at 37°C in a humidified 5% CO_2_ incubator. Pools and clones of MCF10A cells with stable expression of Hemagglutinin (HA)-tagged wt-NTHL1 or HA-tagged R33K-NTHL1 (referred to as dominant lines) were generated as previously described [4]. To generate MCF10A wt-NTHL1 or R33K-NTHL1 pools or clones with stable expression of NTHL1-targeted shRNA constructs, HEK293T cells were transfected with 6 μg of pVSV-G, pRGR, pRSV and pLKO.1 NTHL1 shRNA-15 (CloneID: TRC0000007915) and pLKO.1 NTHL1 shRNA-16 (CloneID: TRC0000007916) (Dharmacon) or pLKO.1 shGFP (Addgene). MCF10A wt-NTHL1 or R33K-NTHL1 expressing cells were infected using virus-containing media collected from the transfected HEK293T cells filtered through a 0.45 μmol/L pore filter and supplemented with 8 μg/mL polybrene. The 6-well plates were centrifuged at 2000 g for 1.5 h. The same procedure as described above was repeated the next day with fresh virus-containing media. Selection for stable expression of the shRNA constructs began 48 h after the second infection using 1 μg/mL puromycin for 8 to 12 days. The resultant pools or clones, referred to wt-shNTH or R33K-shNTH (knockdown pools) were maintained in MCF10A complete growth media with 15 μg/mL hygromycin and 75 ng/mL puromycin.

### Thymine glycol excision assay

The active site fraction of the recombinant wt-NTHL1 and R33K-NTHL1 were equivalent [assessed using a Schiff-base trap assay [26]]. All enzyme concentrations indicated in the text and figure legends refer to the active enzyme concentration. An oligodeoxynucleotide (oligo) (35 bases) with a centrally positioned thymine glycol (ThyGly) was 5ʹ end labeled with ^32^P as previously described [26]. The labeled oligo was annealed to a compliment containing an adenosine opposite the ThyGly. Excision of ThyGly was assayed at 25°C in 100 mmol/L NaCl, 25 mmol/L 4-(2-hydroxyethyl)-1-piperazineethanesulfonic acid (HEPES), pH 8.0, 2 mmol/L ethylenediaminetetraacetic acid (EDTA), and 100 μg/mL bovine serum albumin (BSA). At various time-points aliquots were taken and quenched in 400 mmol/L NaOH and heated to 95°C for 2 min. Two volumes of formamide (96%), 20 mmol/L EDTA was added. Substrate was separated from product using 12% denaturing polyacrylamide gel electrophoresis (PAGE) in 1× Tris/Borate/EDTA (TBE) buffer. Bands were visualized and quantified using the Pharos FX plus Phosphoimager (BioRad). Product was graphed as a function of time and data were fit to a double exponential equation (GraphPad Prism). Enzymes purified from HEK293 cells were quantified by western blot, diluted to equivalent concentration and assayed as above.

### Western blotting

Cells were lysed in modified radioimmunoprecipitation assay (RIPA) buffer (150 mmol/L NaCl, 50 mmol/L Tris pH 7.8, 1% NP-40, 0.25% sodium deoxycholate, protease inhibitors) then centrifuged at 10000 g for 10 min. Cell lysates were combined with 6× loading buffer (375 mmol/L Tris-HCl, 9% sodium dodecyl sulfate (SDS), 50% glycerol, bromophenol blue), separated on a 10% SDS-PAGE gel and transferred to a polyvinylidene difluoride (PVDF-FL) membrane (Millipore, Billerica, MA, USA). The membrane was blocked in Odyssey Casein blocking buffer PBS (Millipore) for 1 hr at room temperature (RT) with gentle shaking. The membrane was then probed with rabbit polyclonal NTHL1 primary antibody (1/1000) (Abcam), mouse monoclonal HA Epitope Tag antibody (1/1000) (Thermo Scientific-Pierce, 2-2.2.14) and mouse monoclonal tubulin primary antibody (1/10000) (Abcam, DM1A) overnight at 4°C with gentle shaking. After 3 washes in PBS/0.1% Tween, the membrane was probed with IRDye 800CW goat anti-mouse IgG (H+L) (1/20000) and IRDye 680RD goat anti-rabbit IgG (H+L) (1/20,000) for 1 h at RT. The membrane was washed with PBS/0.1% Tween and visualized using an Odyssey CLx Infrared Imaging System.

### Cell fractionation

Cytoplasmic and nuclear fractions were isolated from cell lysates using the NE-PER Nuclear and Cytoplasmic Extraction kit (Thermo Scientific) as per the manufacturers’ instructions.

### Measurement of DNA base lesions in wt-NTHL1 and NTHL1 R33K-expressing cells

Genomic DNA was isolated from asynchronous MCF10A wt-NTHL1 or R33K-NTHL1 shNTH-expressing cells either untreated or treated with 10 mmol/L H_2_O_2_ for 30 min at 4°C followed by a 30 min recovery using the Qiagen Blood and Cell Culture DNA Maxi kit without the use of phenol as per the manufacturers’ instructions. The eluted genomic DNA was ethanol-precipitated then washed 3 times with ethanol. Ethanol was removed, and the DNA was dissolved in water for 18 h at 4°C, and then quantified using an absorption spectrophotometer (absorption of 1 = 50 μg DNA). Aliquots (50 μg) of the DNA samples were dried in a SpeedVac under vacuum. DNA samples were dissolved in 50 μL of an incubation buffer consisting of 50 mmol/L phosphate buffer (pH 7.4), 100 mmol/L KCl, 1 mmol/L EDTA and 0.1 mmol/L dithiothreitol. Aliquots of FapyAde-^13^C,^15^N_2_, FapyGua-^13^C,^15^N_2_, 8-OH-Ade-^13^C,^15^N_2_, 8-OH-Gua-^15^N_5_, 5-OH-Cyt-^13^C,^15^N_2_, 5-OH-Ura-^13^C_4_,^15^N_2_, ThyGly-^2^H_4_, 5-OH-5-MeHyd-^13^C,^15^N_2_ and 5,6-diOH-Ura-^13^C,^15^N_2_ (isodialuric acid-^13^C,^15^N_2_) were added as internal standards. Samples were incubated with 1 μg of *E. coli* Nth plus 1 μg of *E. coli* Fpg for 1 h at 37°C in a water bath to release DNA lesions from DNA samples. After incubation, 125 μL of cold ethanol (kept at –20°C) were added to the samples to stop the reaction and to precipitate DNA. The samples were kept at –20°C for 1 h, and then centrifuged at 15000 g for 30 min at 4°C. DNA pellets and supernatant fractions were separated. Ethanol was removed from the supernatant fractions under vacuum in a SpeedVac. Aqueous supernatant fractions were frozen at –80°C for 1 h and then lyophilized to dryness for 18 h. For all measurements, 3 independently prepared DNA samples were used. Dried samples were derivatized and analyzed by gas chromatography-tandem mass spectrometry (GC-MS/MS) as described previously [27].

### Determination of the substrate specificities of wt-NTHL1, R33K-NTHL1 and Δ63-NTHL1

Commercially available calf thymus DNA was dissolved in phosphate buffer (pH 7.4) at a concentration of 0.3 mg/mL. This solution was bubbled with N_2_O for 30 min and irradiated with γ-rays in a ^60^Co γ ray-source at a dose of 10 Gy (dose rate 5.2 Gy/min). Irradiated DNA sample was dialyzed against water at 4°C for 18 h. Water outside the dialysis tube was changed three times during the dialysis. After dialysis, the concentration of DNA in the dialyzed sample was measured by absorption spectrophotometry. Aliquots (50 μg) of the DNA sample were dried in a SpeedVac under vacuum. Unirradiated DNA samples as controls were treated in the same manner. DNA samples were dissolved in 50 μL of an incubation buffer consisting of 50 mmol/L phosphate buffer (pH 7.4), 100 mmol/L KCl, 1 mmol/L EDTA and 0.1 mmol/L dithiothreitol. Aliquots of the stable isotope-labeled internal standards were added as described above. Samples were incubated with 1 μg of wt-NTHL1, 1 μg of R33K-NTHL1, or 1 μg of Δ63-NTHL1 for 1 h at 37°C in a water bath. Three other DNA samples to be used as controls were incubated without any enzyme. After incubation, 125 μL of cold ethanol (kept at –20°C) were added to the samples to stop the reaction and to precipitate DNA. The samples were kept at –20°C for 1 h, and then centrifuged at 15000 g for 30 min at 4°C. DNA pellets and supernatant fractions were separated. Ethanol was removed from the supernatant fractions under vacuum in a SpeedVac. Aqueous supernatant fractions were frozen at –80°C for 1 h and then lyophilized to dryness for 18 h. For all measurements, 3 independently prepared DNA samples were used. Dried samples were derivatized and analyzed by GC-MS/MS as described previously [27].

## CONCLUSIONS

Our study provides evidence that the germline mutation, R33K-NTHL1, induces cellular transformation. Importantly, this study further underscores the importance for investigations into germline mutations in NTHL1 and other BER proteins in order to identify mutations that increase the risk for cellular transformation and may lead to increased cancer incidence in humans harboring these mutations. Ultimately, the dysfunctional phenotypes exhibited by R33K-NTHL1 in this study and in future studies of NTHL1 variants will provide invaluable clues into the broad functions of NTHL1, and more generally BER, in diverse cellular processes.
